# A survey of chiral hypervalent iodine reagents in asymmetric synthesis

**DOI:** 10.3762/bjoc.14.107

**Published:** 2018-05-30

**Authors:** Soumen Ghosh, Suman Pradhan, Indranil Chatterjee

**Affiliations:** 1 Department of Chemistry, Indian Institute of Technology Ropar, Nangal Road, Rupnagar, Punjab 140001, India

**Keywords:** alkene functionalization, asymmetric synthesis, hypervalent iodine, organocatalysis, oxidation

## Abstract

The recent years have witnessed a remarkable growth in the area of chiral hypervalent iodine chemistry. These environmentally friendly, mild and economic reagents have been used in catalytic or stoichiometric amounts as an alternative to transition metals for delivering enantioenriched molecules. Varieties of different chiral reagents and their use for demanding asymmetric transformations have been documented over the last 25 years. This review highlights the contribution of different chiral hypervalent iodine reagents in diverse asymmetric conversions.

## Introduction

It is more than one century ago since the discovery of the first hypervalent iodine reagent (HIR) [1] and hypervalent iodine chemistry has started to flourish as one of the important and leading areas in organic synthesis. In recent years many excellent reviews have detailed the bonding, reactivity, synthesis, and uses of hypervalent iodine reagents [2–14]. These compounds feature a unique three-centered four-electron bond [15–20] that renders them valuable and important alternatives to transition-metal chemistry. Over the last 25 years hypervalent iodine reagents have gained growing application due to their reduced toxicity, ready availability and lower costs as replacement for transition metals leading to several “metal-free” like chemical transformations.

The ongoing demand of modern synthetic chemistry for the development of catalytic enantioselective C–C bond formation reactions turned the attention of the scientific community towards the evolution of new chiral hypervalent iodine reagents. In recent years, many complex synthetic challenges have been successfully addressed by applying these reagents [21–22]. The superior advantage of these reagents lies in their strong electrophilicity and appreciable oxidizing properties. The transformations associated with asymmetric induction mainly focused on the asymmetric oxidation and oxidative dearomatization chemistry. Asymmetric difunctionalization of alkenes, α-functionalization of carbonyls and also some typical 1,2-aryl rearrangement reactions add further value to this chemistry.

**Scheme 1:**
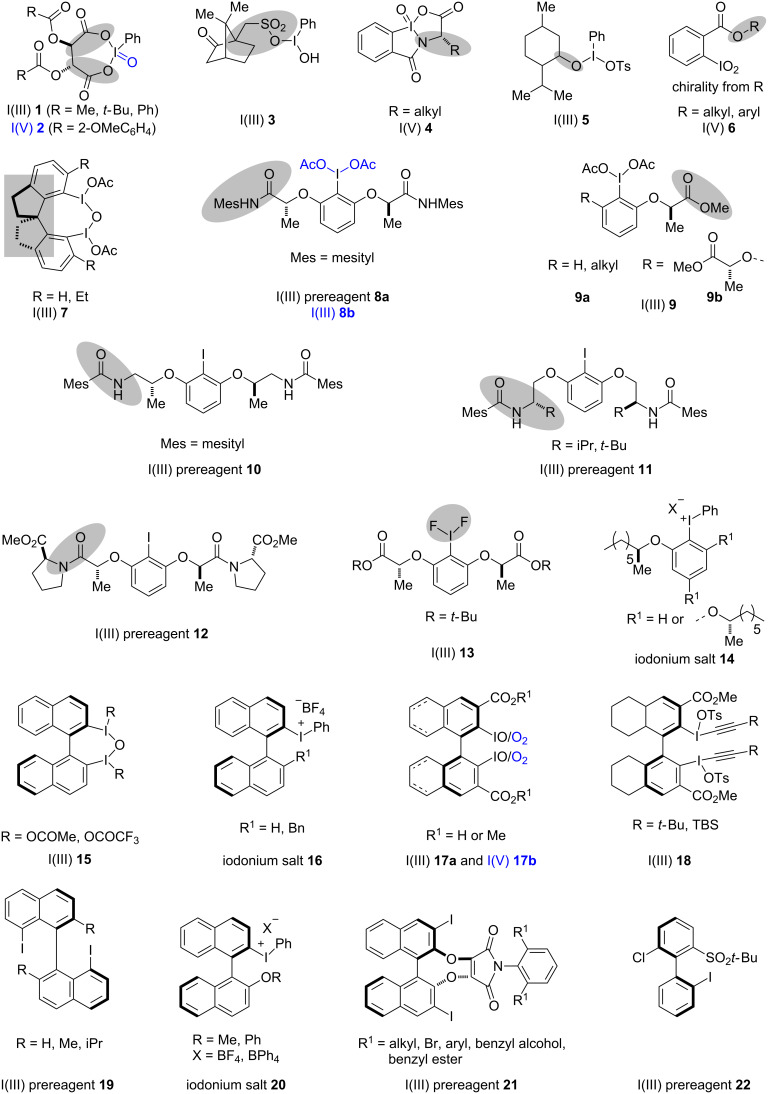
An overview of different chiral iodine reagents or precursors thereof.

The strategies used for the synthesis of chiral hypervalent iodine reagents include either the introduction of chirality through the attachment of chiral acids or chiral alcohols to the iodine centers by ligand exchange or are achieved by the introduction of axial chirality through the iodoarene backbone. A series of chiral iodine reagents are documented below (Scheme 1). In many cases chiral I(I) reagents get oxidized in situ to the hypervalent I(III) reagents and/or these chiral I(III)/I(V) reagents are used in a catalytic amount in the presence of an external oxidant. The use of catalytic chiral hypervalent iodine reagents in asymmetric catalysis is one of the most challenging ongoing topics and this review will focus on the development of various chiral hypervalent iodine reagents and their application in typical organic transformations.

## Review

### Asymmetric oxidation of sulfides

**Scheme 2:**
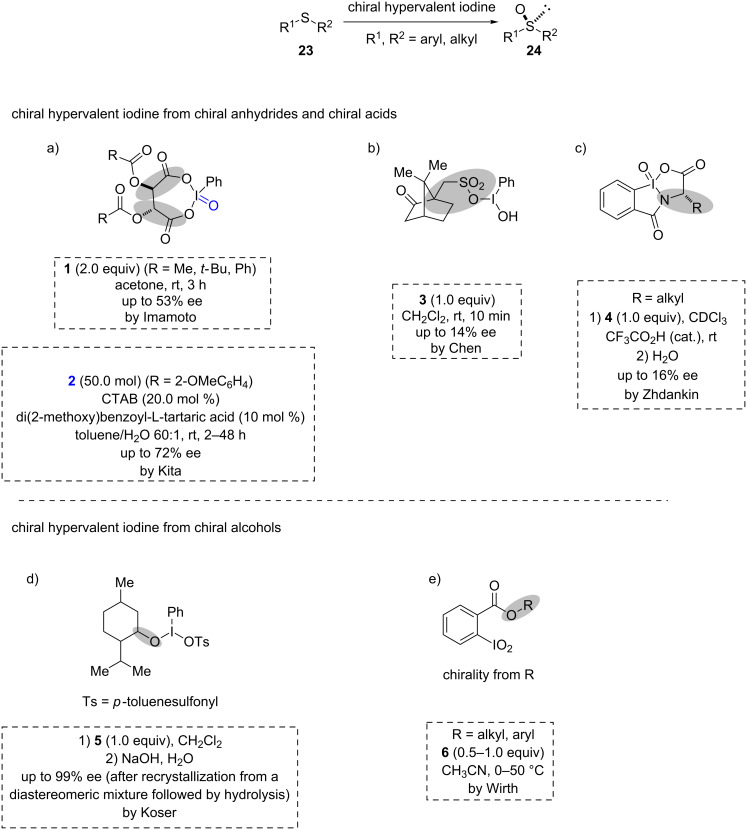
Asymmetric oxidation of sulfides by chiral hypervalent iodine reagents.

Pribam was the first to use chiral iodine reagents [23]. After a long time without further developments in this direction, Imamoto et al. introduced a new class of chiral hypervalent iodine reagents **1** obtained by the reaction of iodosylbenzene with various derivatives of L-tartaric acid anhydrides in 1986. A promising asymmetric induction was achieved for the oxidation of sulfides **23** to sulfoxides **24**. This marked the beginning of an era of asymmetric oxidation of sulfides. However, the presence of *C*_2_ symmetry in the chiral unit is essential to obtain decent enantioselectivity [24]. Later, Kita et al. used chiral tartaric acid derivatives to synthesize chiral I(V) reagents **2** from PhIO_2_. This represented the first example of the catalytic use of chiral hypervalent reagents in the oxidation of sulfides to sulfoxides with decent enantioselectivities (ees, Scheme 2a). The asymmetric oxidations were examined in 20 mol % cetyltrimethylammonium bromide (CTAB) reversed micelles [25]. Interestingly, Varvoglis et al. synthesized another new class of a chiral reagent **3** using (+)-camphor sulfonic acids as the source of chirality [26] which was used by Chen et al. for the oxidation of sulfides to sulfoxides with good yields but with poor enantioselectivity (Scheme 2b) [27]. Later, Zhdankin et al. synthesized different classes of chiral I(V) reagents **4** based on various amino acids as sources of chirality. The oxidation of the readily available 2-iodobenzamides (synthesized from amino acid derivatives) with potassium bromate or Oxone (2KHSO_5_/KHSO_4_/K_2_SO_4_) efficiently delivered the I(V) reagents **4** (Scheme 2c) [28–29]. Although good product yields were obtained for the oxidation of sulfides, the ees were very low.

New classes of chiral hypervalent iodine reagents were obtained by the introduction of chiral alcohols directly to the iodine reagent through ligand exchange. Koser et al. used (+)- or (−)-menthol as a source of chirality during their synthesis of the new chiral iodine reagent **5** [30]. Chiral sulfoxides **24** (having R^1^ = *p*-Tol, *t-*Bu, Bn and R^2^ = Me) were obtained for the first time with excellent enantioselectivity (Scheme 2d). Another class of chiral I(V) reagents **6** was synthesized by Wirth et al. who synthesized the desired compounds through esterification between chiral alcohols and the I(I)-substituted aromatic acids followed by oxidation with dimethyldioxirane (Scheme 2e) [31]. A summary of chiral hypervalent iodine reagents used in the asymmetric oxidation of sulfides is sketched below (Scheme 2).

### Asymmetric oxidative dearomatization, alkene functionalization and rearrangement strategy

#### Oxidative dearomatization

**Scheme 3:**
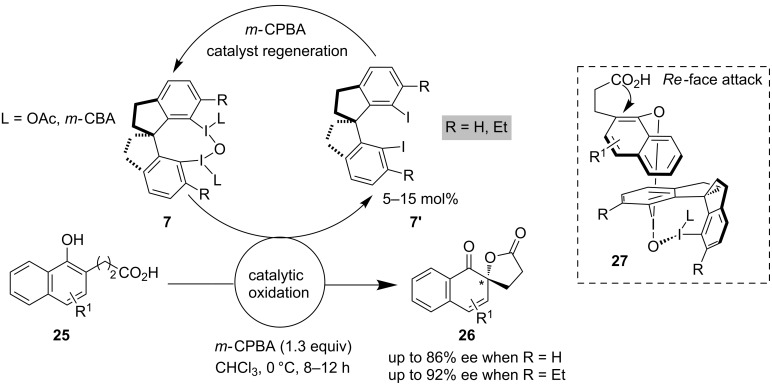
Oxidative dearomatization of naphthol derivatives by Kita et al.

Asymmetric oxidative dearomatizations and the use of dearomatized products to generate chiral complex molecular scaffolds in a short and efficient way is one of the attractive strategies used in chiral hypervalent iodine chemistry. Kita et al. for the first time developed a new chiral I(III) catalyst **7** having a rigid spirocyclic backbone. They applied it for the enantioselective oxidative dearomatization of phenolic derivatives **25** (spirolactonization) which is known as Kita oxidation to yield spirocyclic compounds **26** with good enantioselectivity [32]. The indication of an associative mechanism was also confirmed due to an increased enantioselectivity observed in polar solvents. Further, they were able to improve the enantioselectivity by implying steric effects at the *ortho*/*ortho*′ (R = Et in **7**) positions of the aromatics (Scheme 3) [33]. The regeneration of the catalyst was achieved by *m*-CPBA converting iodine compound **7′** to chiral catalyst **7**. The authors predicted a plausible mechanism and transition-state model **27** for the formation of the major isomer through the attack of the carboxylic acid group to the *ipso* position of the naphthol ring from the less sterically hindered *Re*-face of the substrate **25**. It is worth mentioning that very recently they have introduced a new kind of binaphthyl-based chiral I(III) prereagent **19** with the 8 and 8′ positions of the naphthalene substituents being occupied by iodide. Here they have observed that this chiral hypervalent iodine reagent **19** in the presence of co-oxidant *m*-CBPA is very useful for the dearomatizing spirocyclization of naphthol carboxylic acid [34].

**Scheme 4:**
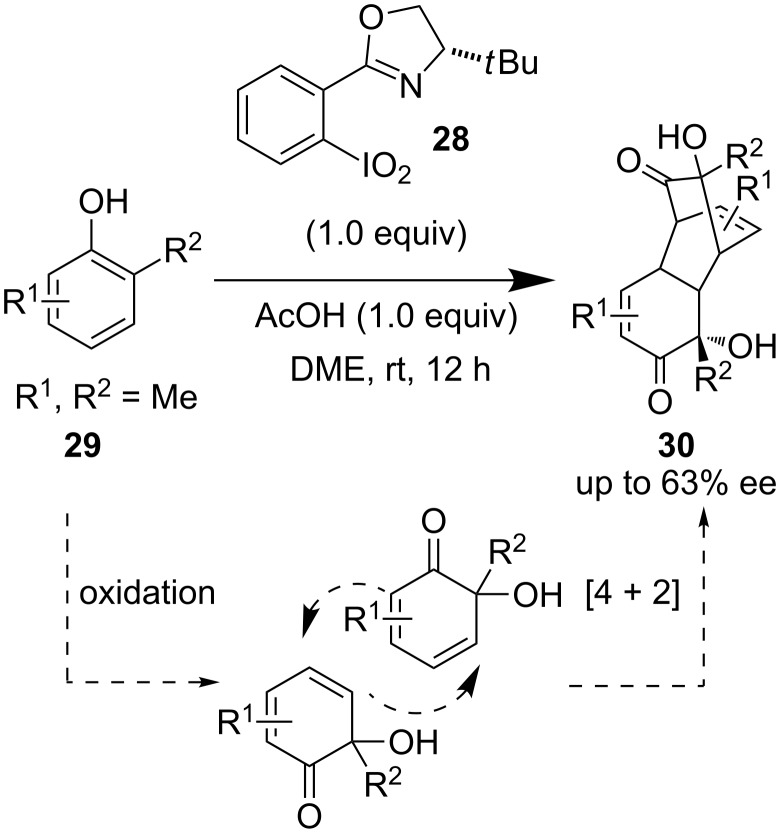
[4 + 2] Diels–Alder dimerization reported by Birman et al.

Later Birman et al. reported a new variation of a chiral I(V) reagent, namely 2-(*o*-iodoxyphenyl)oxazoline derivative **28** [35]. The reagent was applied to an asymmetric [4 + 2] Diels–Alder dimerization of phenolic derivatives **29** to construct tricyclic derivatives **30** with moderate enantioselectivity (Scheme 4). Although modest ees were obtained, this chiral oxazoline-based compound demonstrated encouraging potential as a new class of chiral hypervalent iodine reagent.

**Scheme 5:**
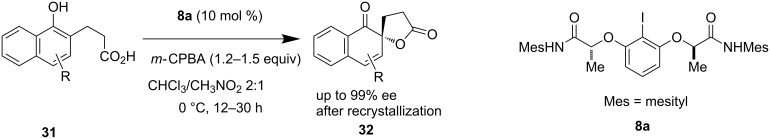
*m-*CPBA guided catalytic oxidative naphthol dearomatization.

Fujita et al. synthesized non *C*_2_-symmetric chiral iodoarene reagents **9a** derived from lactic acid derivatives and utilized them in stoichiometric fashion for the synthesis of chiral tetrahydrofuran derivatives [36]. A major breakthrough was achieved in this field by the discovery of a family of conformationally flexible *C*_2_-symmetric chiral iodoarene reagents. Ishihara et al. thoughtfully designed a new class of *C*_2_-symmetric chiral iodoarene precatalyst **8a** using (−)-ethyl lactate as a chiral linker with the aromatics attached followed by its successful conversion to the amide derivative to generate precatalyst **8a** [37–38]. Application of this precatalyst **8a** was employed for the Kita oxidation [32] with a high level of enantioselectivity. Naphthol derivatives **31** were converted to spirocyclic lactones **32** in the presence of *m-*CPBA as co-oxidant for the in situ generation of the I(III) catalyst. Secondary n–π* and/or hydrogen-bonding interactions of the catalyst ensured remarkable enantioselectivities (Scheme 5).

**Scheme 6:**
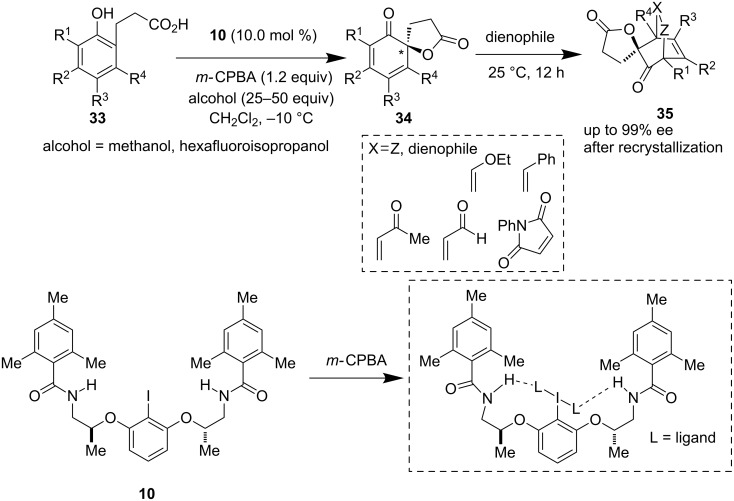
Oxidative dearomatization of phenolic derivatives by Ishihara et al.

Later, this dearomatization strategy was further reinvestigated by the Ishihara group using a new chiral iodine precatalyst **10** derived from a chiral 2-aminoalcohol [39]. Its application in the oxidative dearomatization of phenol **33** and the subsequent reaction of the so-obtained dienes **34** with different dienophiles furnished Diels–Alder adducts **35** with excellent enantioselectivity. Intramolecular H-bonding and the presence of an achiral alcohol as additive helped them to achieve outstanding enantioselectivity (up to 99%) even when using very low catalyst loadings (1–10 mol %, Scheme 6)

**Scheme 7:**
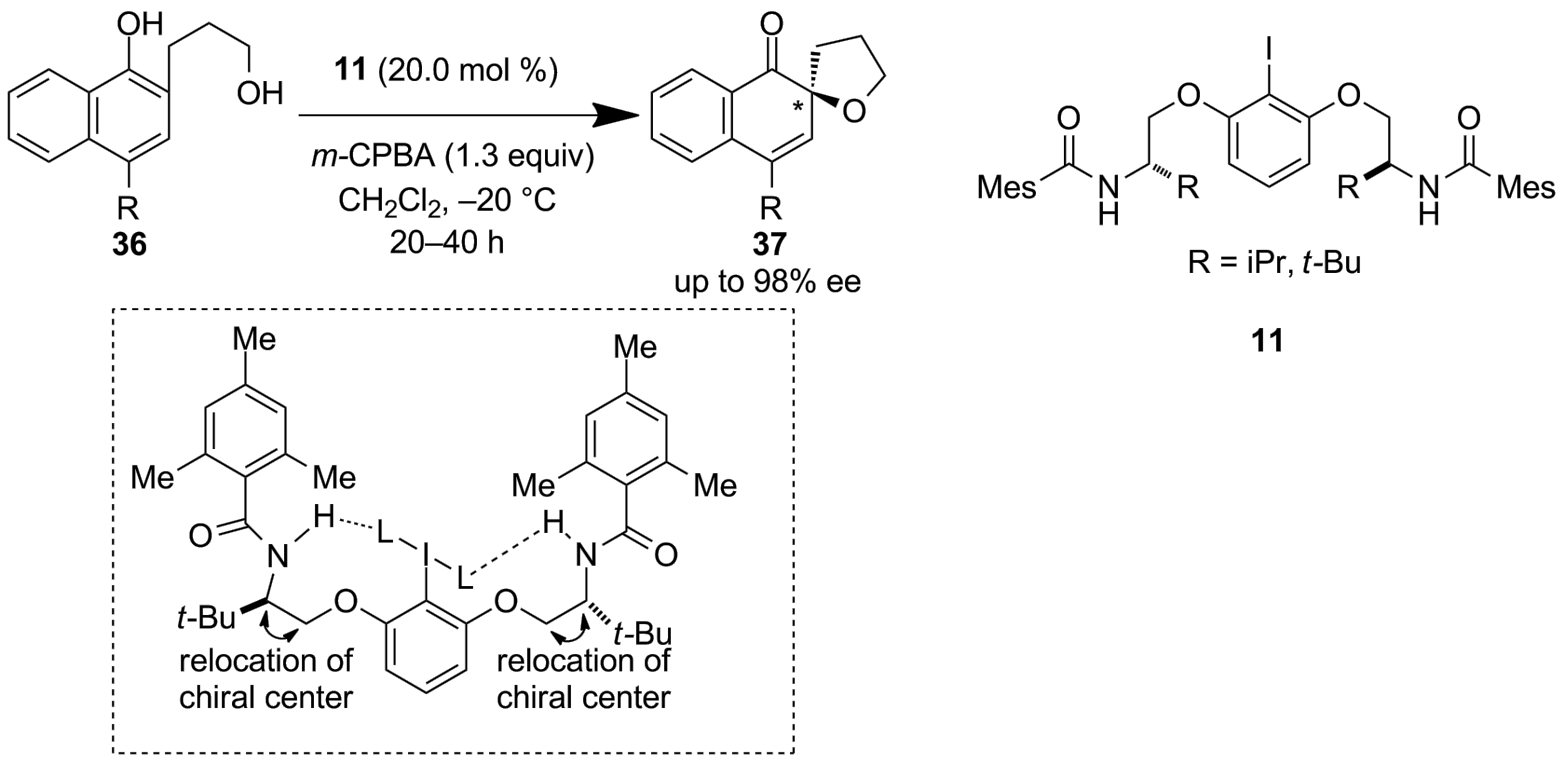
Oxidative spirocyclization applying precatalyst **11** developed by Ciufolini et al.

Ciufolini et al. further carefully modified precatalyst **10** to generate a new chiral iodine precatalyst **11** [40]. They critically altered the chiral center next to the amide NH group to achieve a high catalyst efficiency (Scheme 7). The crucial point is the better hydrogen-bonded conformation of **11** which imparted superior reactivity compared to Ishihara’s system without the need of achiral alcohols as an additive. Profitable results were obtained regarding the oxidative cyclization of phenolic derivatives **36** to spirocyclic compounds **37**.

**Scheme 8:**
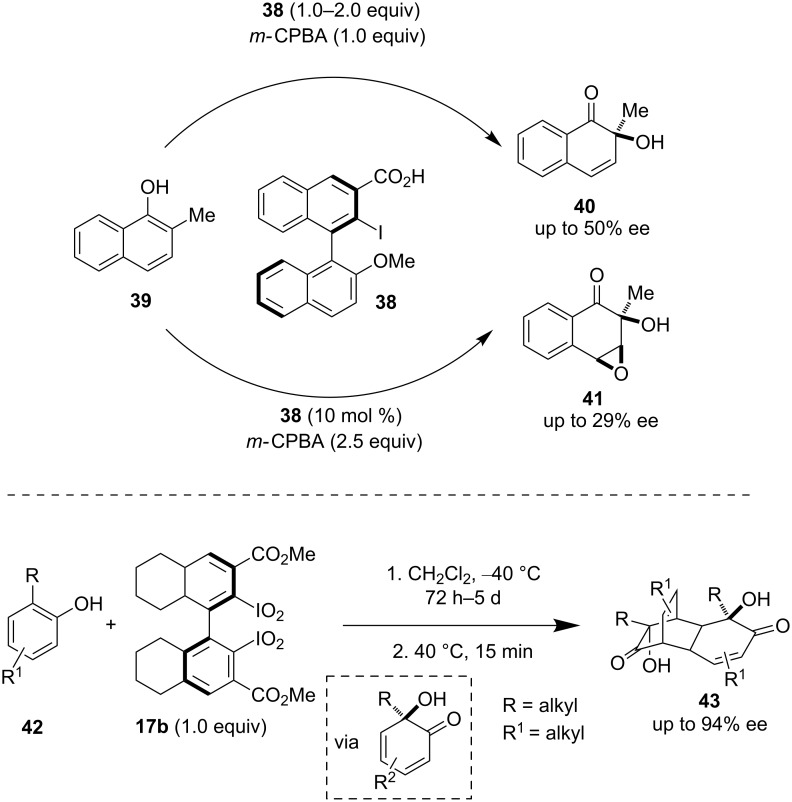
Asymmetric hydroxylative dearomatization.

Chiral hypervalent iodine reagents having binaphthyl backbones were used by Quideau et al. for the α-hydroxylation of phenolic derivatives via oxygenative dearomatization. Quideau et al. showed that iodobiarene **38** was oxidized in situ by *m-*CPBA to generate the I(III) reagent which is responsible for the hydroxylative naphthol dearomatization affording the product in moderate enantioselectivity (Scheme 8 upper part). By this method naphthol **39** could be oxidized to chiral *o*-quinol **40** with 50% ee [41]. Varying the catalyst loading could alter the reaction outcome to afford either *o*-quinol **40** or epoxy *o*-quinol **41**.

Recently, Pouységu and Quideau et al. modified their iodobiarenes to synthesize a new class of I(III) and I(V) reagents **17**. These were applied for the hydroxylative dearomatization of phenolic derivatives **42** followed by the successive use of the hydroxylated products as dienes in [4 + 2] cycloaddition reactions [42]. This new reagent promoted oxygen transfer in phenol dearomatization, leading to the formation of cyclodimerization products **43** with high enantioselectivity (up to 94% ee, Scheme 8 lower part).

#### Alkene functionalization

**Scheme 9:**
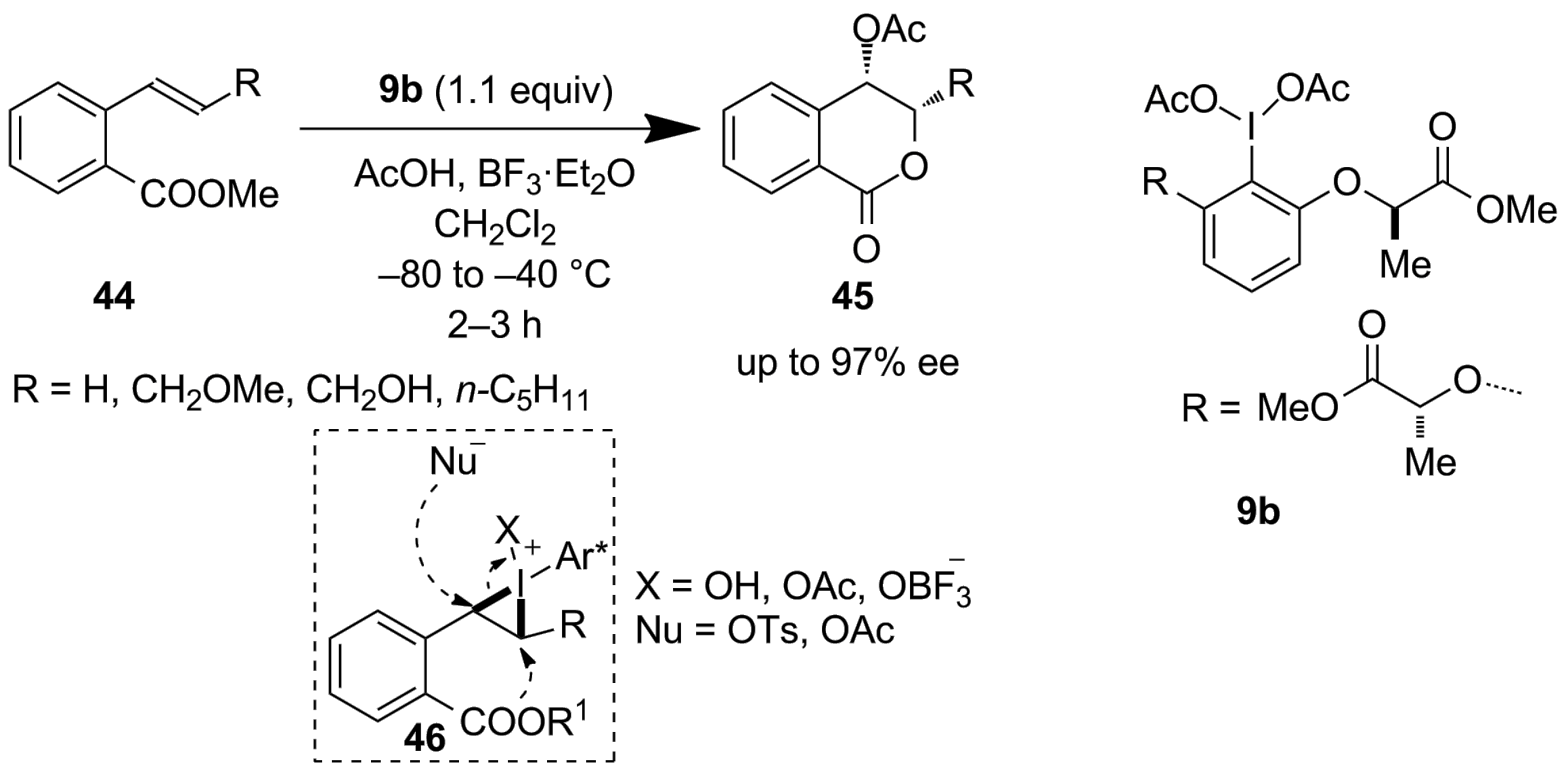
Enantioselective oxylactonization reported by Fujita et al.

Nearly simultaneously to Ishihara’s work, Fujita et al. reported on the modification of their previously synthesized non *C*_2_-symmetric reagent **9a** to obtain a *C*_2_-symmetric chiral iodoarene reagent **9b** having an ester end group instead of an amide (as in case of Ishihara’s work). The enantioselective oxylactonization was achieved efficiently using stoichiometric amounts of chiral reagent **9b** (Scheme 9). This lactate-derived I(III) reagent **9b** was used successively for the synthesis of δ-lactones **45** in a highly stereoselective manner starting from **44** [43]. The formation of cyclic iodonium **46** is the vital part of this difunctionalization process.

**Scheme 10:**
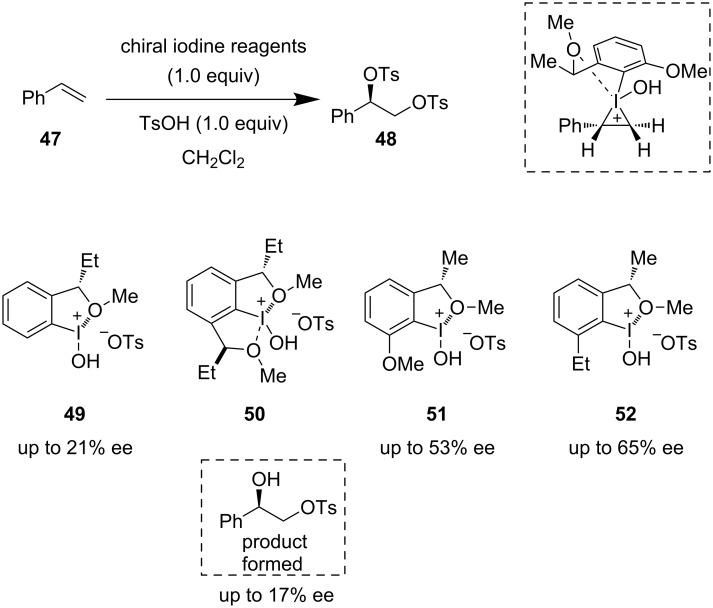
Dioxytosylation of styrene (**47**) by Wirth et al.

Wirth et al. were the first to introduce asymmetric dioxytosylation of styrene (**47**) using a new class of chiral hypervalent iodine reagents **49**–**52** to furnish **48** with moderate enantioselectivity (Scheme 10) [44–47]. Their constant efforts towards alkene dioxygenation helped them to discover new chiral hypervalent iodine reagents and also to reach up to 65% enantioselectivity using **52**.

**Scheme 11:**
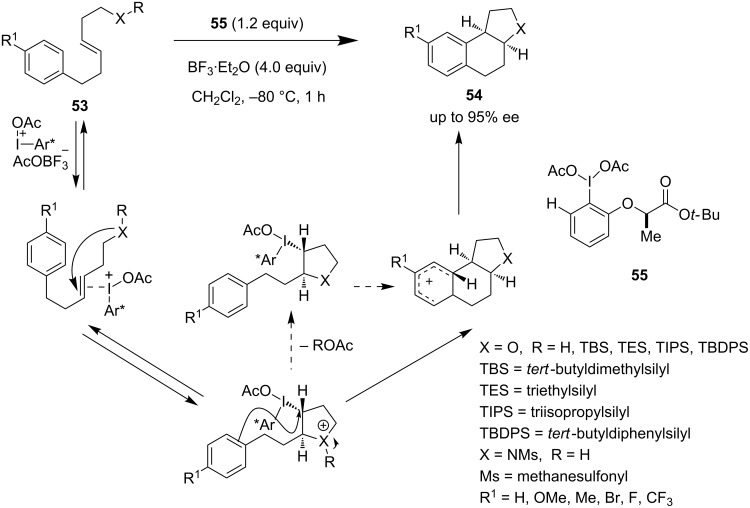
Oxyarylation and aminoarylation of alkenes.

Fujita et al. further explored the difunctionalization strategy for the development of diacetoxylation of alkenes following a Prevost and Woodward reaction [48]. Recently, the same group used chiral iodine reagent **55** together with acid co-reagent for the intramolecular oxyarylation and aminoarylation of alkenes **53** to produce **54** (Scheme 11). The presence of a silyloxy group is essential to achieve high enantioselectivity in case of the oxyarylation [49]. The Lewis acid activates the hypervalent chiral iodine reagent and then adds to the alkene system. The nucleophilic addition of the internal oxy/amino group followed by the nucleophilic addition of the aryl group delivers the desired products **54**. The key to success also lies on the enantiotopic face discrimination of the alkene by the lactate-based chiral iodine reagent.

**Scheme 12:**
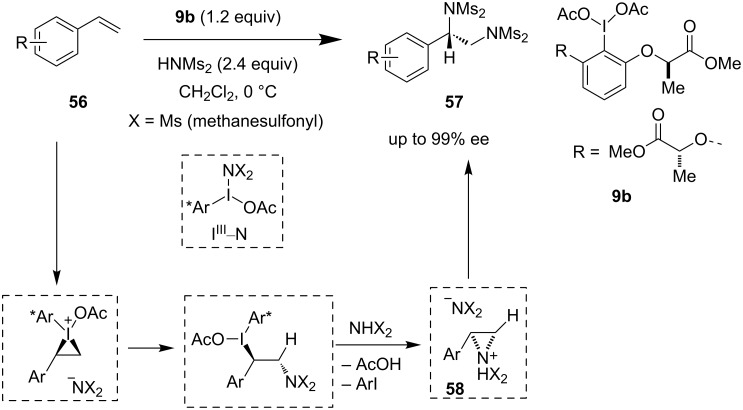
Asymmetric diamination of alkenes.

This difunctionalization strategy was further showcased by Muñiz et al. as intermolecular diamination protocol of alkenes **56** using **9b** (Scheme 12) [50]. This represented the first example of an asymmetric diamination of simple nonfunctionalized alkenes to acquire diaminated products **57**. The existence of an I(III)–N bond under ligand exchange conditions and the formation and ring opening of aziridinium intermediate **58** elucidate the product formation in this transformation [51].

**Scheme 13:**
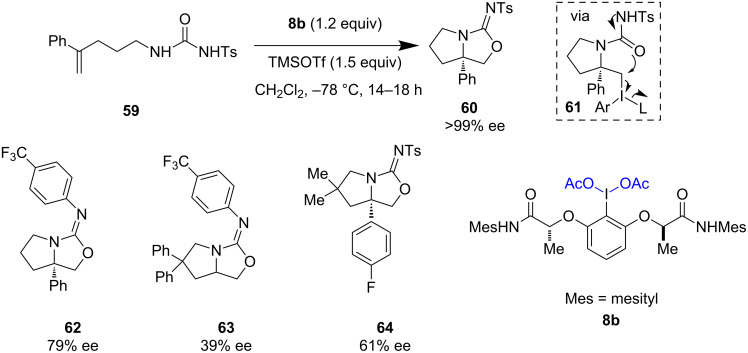
Stereoselective oxyamination of alkenes reported by Wirth et al.

Wirth et al. successfully employed I(III) reagent **8b** in combination with trimethylsilyltriflate (TMSOTf) for the stereoselective oxyamination of **59** to furnish isourea **60** with >99% ee (Scheme 13) [52]. Both the Lewis acid and solvent used play an important role in this transformation. This method was applied to the synthesis of other isourea derivatives **62**–**64** with moderate enantioselectivity. The reactions were triggered by the activation of olefins followed by the formation of C–N bonds. The subsequent intramolecular substitution reaction of intermediate **61** having hypervalent iodine as a good leaving group yielded the required heterocycles.

**Scheme 14:**
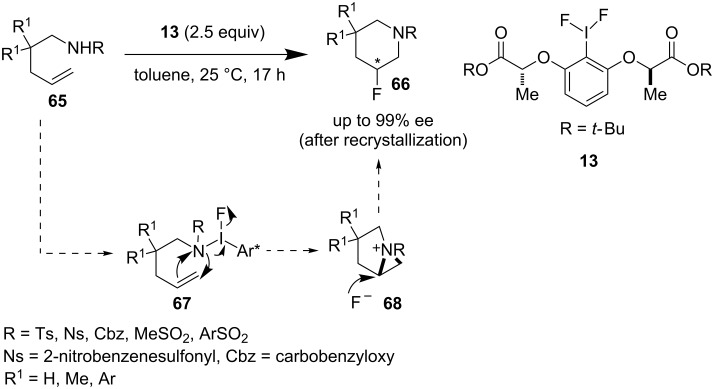
Enantioselective and regioselective aminofluorination by Nevado et al.

After Wirth’s report, Nevado et al. discovered a newly modified chiral iodine reagent **13** analogous to lactate-based chiral iodoarenes [53]. They have utilized this chiral difluoroiodonium salt **13** for the asymmetric synthesis of aminofluorinated compounds **66** from **65** (Scheme 14). In addition to this, they extended this methodology for the regioselective intermolecular aminofluorination of styrenes with a racemic catalyst. The nucleophilic attack of the nitrogen atom onto the alkene (intermediate **67**) to generate aziridinium ion **68** is the crucial step in this transformation.

**Scheme 15:**
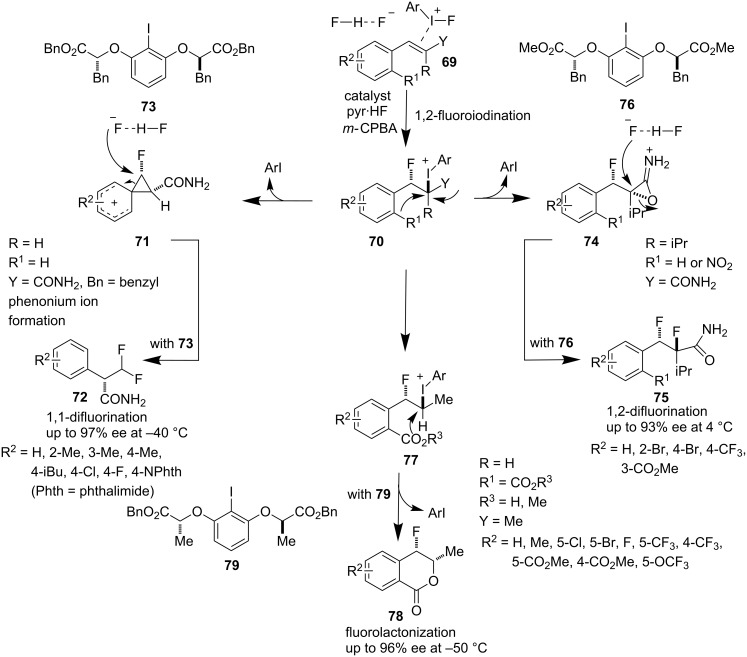
Fluorinated difunctionalization reported by Jacobsen et al.

Recently, Jacobsen et al. developed a highly stereoselective difunctionalization method for the synthesis of chiral fluorine-containing molecules and the 1,2-difluorination, 1,1-difluorination and fluorolactonization protocols appeared almost simultaneously (Scheme 15). The lactate-based *C*_2_-symmetric chiral iodine precatalysts **73**, **76**, and **79** were used to deliver chiral fluorinated scaffolds from alkene starting materials **69** in the presence of pyr**^.^**HF as a nucleophilic fluoride source. The reactions were guided by the formation of intermediate **70**. Anchimeric assistance via the phenonium ion intermediate **71** and subsequent ring-opening rearrangement delivered the 1,1-difluorinated products **72** in the presence of catalyst **73** [54]. On the other hand the anchimeric assistance via participation of the amide carbonyl group (intermediate **74**) dictated the formation of 1,2-difluorinated products **75** and catalyst **76** was identified as the optimal catalyst for this transformation. The 1,2-difluorinated products **75** can also be obtained with high diastereoselectivity by an anchimeric assistance of an *o*-NO_2_ group present in the aryl ring [55]. The authors cleverly replaced the *o*-NO_2_-substituent with a CO_2_R group (R = H or Me). With this modification they were able to obtain fluorolactonization products with high enantioselectivity using **79** as a catalyst, via the intramolecular displacement of the aryl iodide by the CO_2_R group in **77** leading to chiral lactones of type **78** [56].

#### Rearrangement strategy

**Scheme 16:**
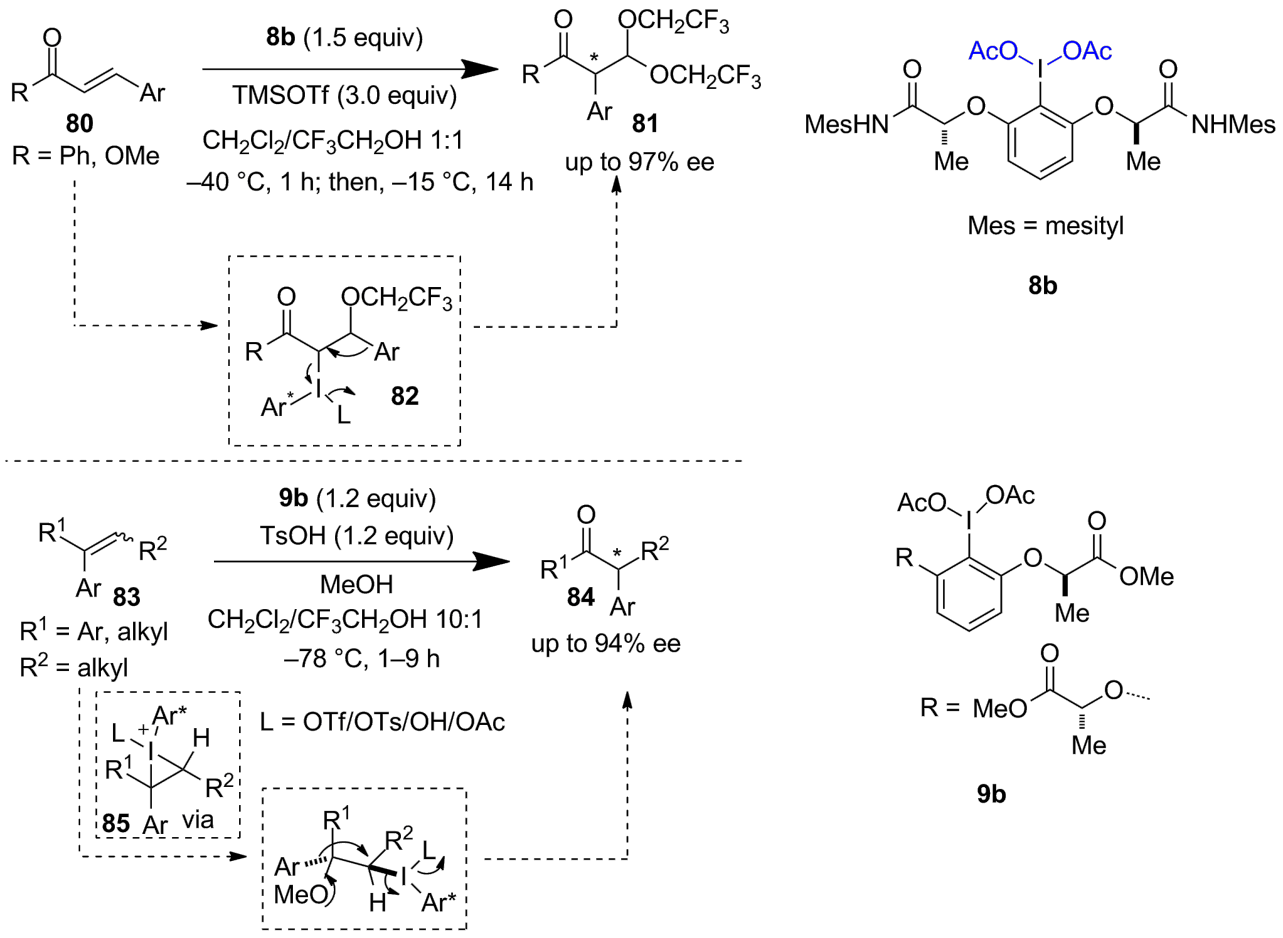
Aryl rearrangement reported by Wirth et al.

Wirth et al. used I(III) reagent **8b** for the development of a stereoselective oxidative rearrangement method to synthesize α-arylated carbonyls **81** from α,β-unsaturated carbonyls **80** (Scheme 16, upper part) [57–58]. The reaction proceeds via the formation of the phenyliodinate intermediate **82** followed by a stereoselective 1,2-aryl migration. Elegantly, they utilized the 1,2-aryl migration approach to develop an enantioselective oxidative rearrangement of 1,1-disubstituted olefins **83** leading to the formation of valuable α-arylated ketones **84**. In this reaction I(III) reagent **9b** gave the best reaction outcome. Key to the success of the reaction is the formation of the cyclic iodonium ion intermediate **85** (Scheme 16, below part) [59–60].

### Asymmetric α-functionalization strategy

Methods for carbonyl α-functionalizations are still considered as highly demandable in synthetic organic chemistry. In this regard transition metals have been successfully applied and even allow accomplishing such transformations asymmetrically. On the other hand, diaryliodonium salts are known to transfer aryl groups ultimately leading to α-arylated products. This part of the review focuses on the development of α-functionalization strategies based on chiral diaryliodonium reagents having either an axially chiral backbone or that can be considered analogous to the *C*_2_-symmetric iodoarene moiety. For this purpose various chiral iodine reagents were synthesized having an axially chiral biaryl backbone. In this part, we mainly focused on the transformations using chiral iodine reagents instead of achiral iodine reagents in a combination with other chiral sources [61].

**Scheme 17:**
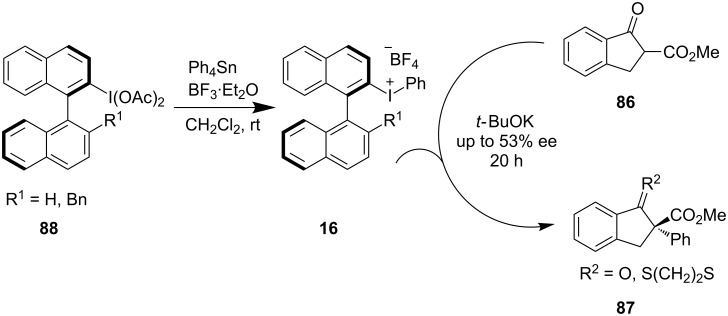
α-Arylation of β-ketoesters.

More than one century after Pribam discovered diphenyliodonium tartrate [23], Ochiai et al. realized the introduction of chirality through incorporation of binaphthyl backbones [62] and they synthesized new classes of chiral hypervalent iodine reagents **15**. Later, to ensure asymmetric transformations, the same group developed the synthesis of more effective chiral iodonium salts **16** which were used for the α-arylation of β-ketoester **86** to deliver α-arylated β-ketoesters **87** with moderate enantioselectivity (Scheme 17) [63]. This was the first example of an asymmetric α-arylation of β-ketoesters using hypervalent iodine reagents. A more reactive organostannane derived Sn–I(III) exchange in the presence of BF_3_**·**Et_2_O was the crucial step in the synthesis of the chiral iodonium salts from **88**.

In view of developing asymmetric α-arylations of carbonyls, Olofsson et al. has independently synthesized a new class of diaryliodonium salts **14** with different stereoelectronic properties by using aliphatic alcohols as a sole source of chirality [64]. Olofsson and Wirth et al. also jointly reported the synthesis of new structurally distinct chiral reagents **20** considering their interest towards asymmetric metal-free arylation [65].

**Scheme 18:**
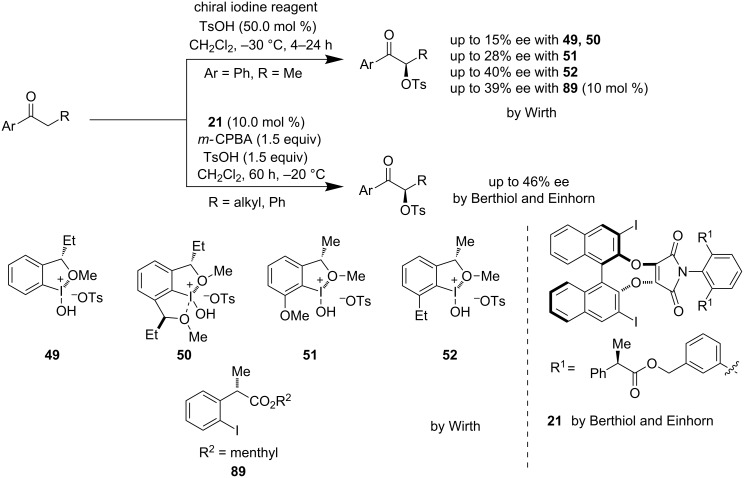
Asymmetric α-oxytosylation of carbonyls.

In 1997, Wirth et al. for the first time reported an asymmetric α-oxytosylation of propiophenone using hypervalent iodine reagents **49**/**50** [45]. Later they improved the enantioselectivity by a structurally modified catalyst **51** to obtain up to 28% ee [46]. After a further few years, in 2001, they came up with a modified catalyst **52** which allowed them to reach up to 40% ee [47]. A catalytic variant of this methodology was developed by the same group using *m-*CPBA as co-oxidant together with catalyst **89** to get up to 39% ee [66] (Scheme 18).

In 2013, Berthiol and Einhorn et al. demonstrated an intermolecular asymmetric α-oxytosylation of ketones by using a new family of chiral hypervalent iodine catalyst **21** with up to 46% ee [67]. The investigation of the reaction mechanisms revealed that the steric crowding around the iodine center improves the enantioselectivity (Scheme 18).

**Scheme 19:**
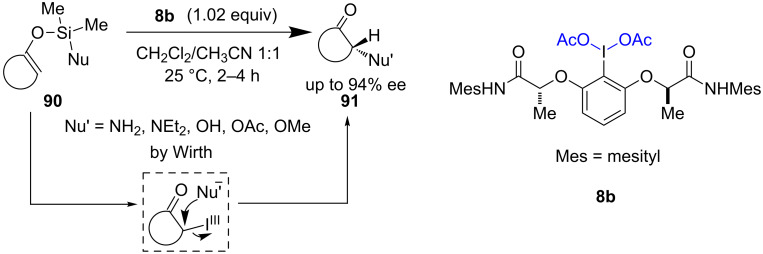
Asymmetric α-oxygenation and α-amination of carbonyls reported by Wirth et al.

Asymmetric oxygenation and nitrogenation reactions of carbonyls were established by Wirth et al. Nucleophile transfer from silyl enol ethers **90** delivered α-functionalized carbonyls **91** with good enantioselectivity [68]. “Umpolung” reactivity and silyl-tethered enol ethers allowed the delicate synthesis of α-functionalized carbonyls (Scheme 19). *C*_2_-symmetric I(III) reagent **8b** was used to obtain high enantioselectivity.

**Scheme 20:**
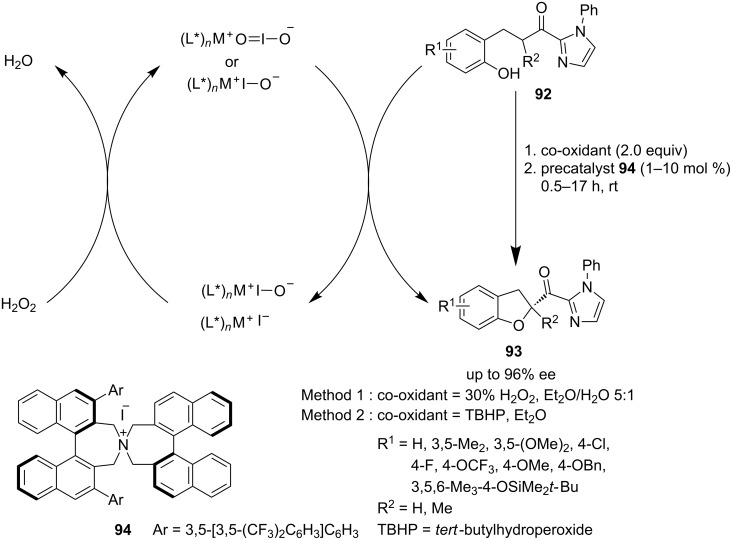
Asymmetric α-functionalization of ketophenols using chiral quaternary ammonium (hypo)iodite salt reported by Ishihara et al.

Ishihara et al. appealingly reported an oxidative cycloetherification of ketophenols **92** in the presence of an in situ generated chiral quaternary ammonium (hypo)iodite salt **94**, with hydrogen peroxide as an oxidant to deliver chiral dihydrobenzofuran derivatives **93** as α-functionalized products of ketophenols **92** (Scheme 20) [69]. The substituents at the 3,3′-position of the binaphthyl moiety of the salt **94** played a crucial role to achieve high enantioselectivities up to 96%.

**Scheme 21:**
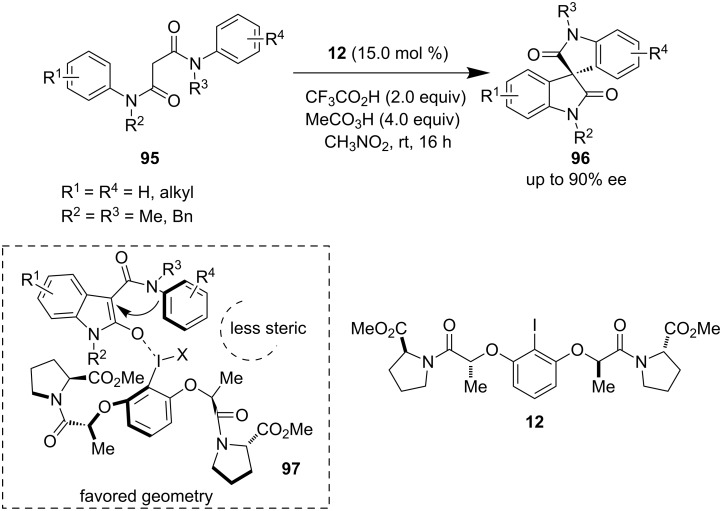
Oxidative Intramolecular coupling by Gong et al.

Very recently, Gong et al. developed an asymmetric oxidative intramolecular cross-coupling of C−H bonds in **95** using catalytic chiral iodine **12** for the synthesis of a diverse array of spirooxindoles **96**. Ishihara’s catalyst was modified by using an (*S*)-proline derivative to achieve a high level of enantioselectivity in the presence of peracetic acid (Scheme 21) [70]. They postulated the formation of possible intermediate **97** which favored the nucleophilic attack of the aryl ring from the less sterically hindered side. Later, Du et al. used this same precatalyst **12** to obtain spirofurooxindole derivatives with high enantioselectivity through cascade cross-coupling sequences [71].

**Scheme 22:**
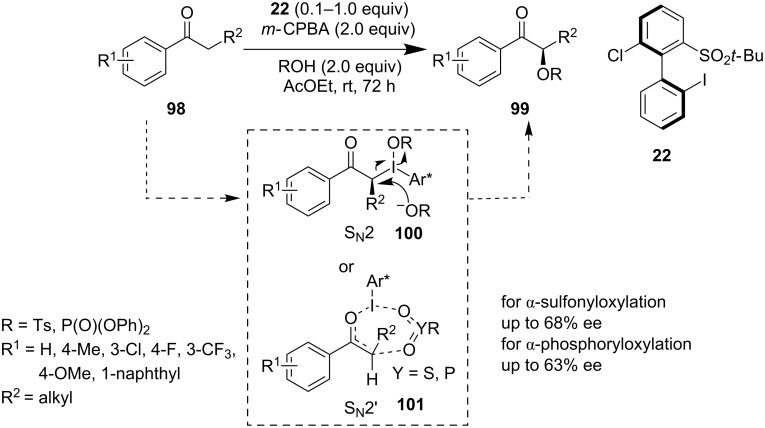
α-Sulfonyl and α-phosphoryl oxylation of ketones reported by Masson et al.

Latterly, Masson et al. reported a new chiral iodoarene prereagent **22** which they have used for the direct oxygenation of carbonyls **98**. They were able to get α-sulfonyls and α-phosphoryl oxyketones **99** with moderate ees (Scheme 22) [72]. A new type of non *C*_2_-symmetric chiral hypervalent reagent was utilized for the asymmetric α-oxygenation of carbonyls. Nucleophilic attack of the oxygen nucleophile to the intermediate **100** or alternatively a reaction pathway through *O*-enolate intermediate **101** can explain the desired product formation.

**Scheme 23:**
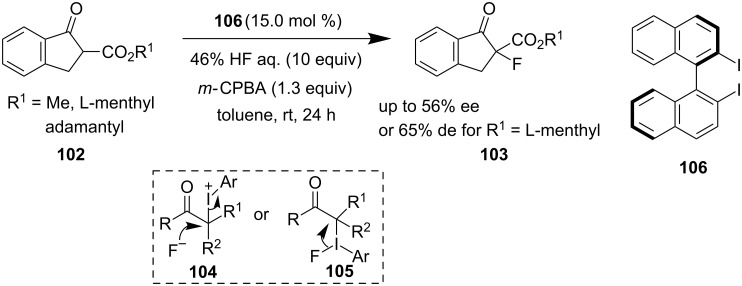
α-Fluorination of β-keto esters.

In 2014, Kita and Shibata reported a catalytic, enantioselective, nucleophilic fluorinating technique of β-keto esters **102** using **106**/HF/*m-*CPBA as a catalytic system to access fluorinated β-keto esters **103** with moderate enantioselectivity [73]. β-Keto esters having sterically hindered adamantyl or menthyl groups lead to good selectivity. However, no further enhancement of ee could be achieved even by using a 50 mol % catalyst loading. A nucleophilic attack of the fluoride ion to the intermediate **104** or a possible ligand coupling pathway via **105** could justify the product formation (Scheme 23).

**Scheme 24:**
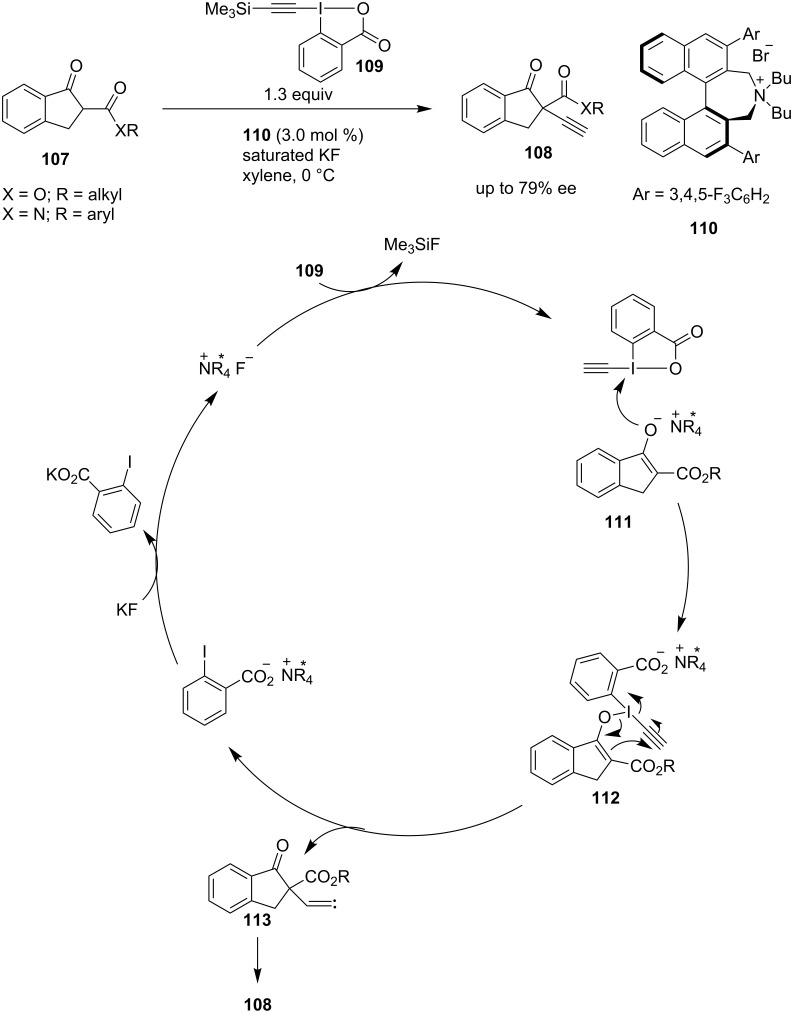
Alkynylation of β-ketoesters and amides catalyzed by phase-transfer catalyst.

Waser et al. developed an asymmetric alkynylation of β-ketoesters and amides **107** catalyzed by a phase-transfer catalyst [74]. Their previous findings on the same reaction using a Cinchona-based phase-transfer catalyst [75] was further improved by using Maruoka’s binaphthyl-derived ammonium salt **110**. The formation of intermediate **112** (chiral catalyst still attached to the substrate) from the enolate intermediate **111** followed by the generation of a C**–**C bond via conjugate addition delivered intermediate carbene **113**. A 1,2-hydrogen shift led to the formation of products **108** with enantioselectivities up to 79% (Scheme 24). Later, Maruoka et al. improved the enantioselectivity up to 95% ee for the alkynylation of β-ketoesters [76].

**Scheme 25:**
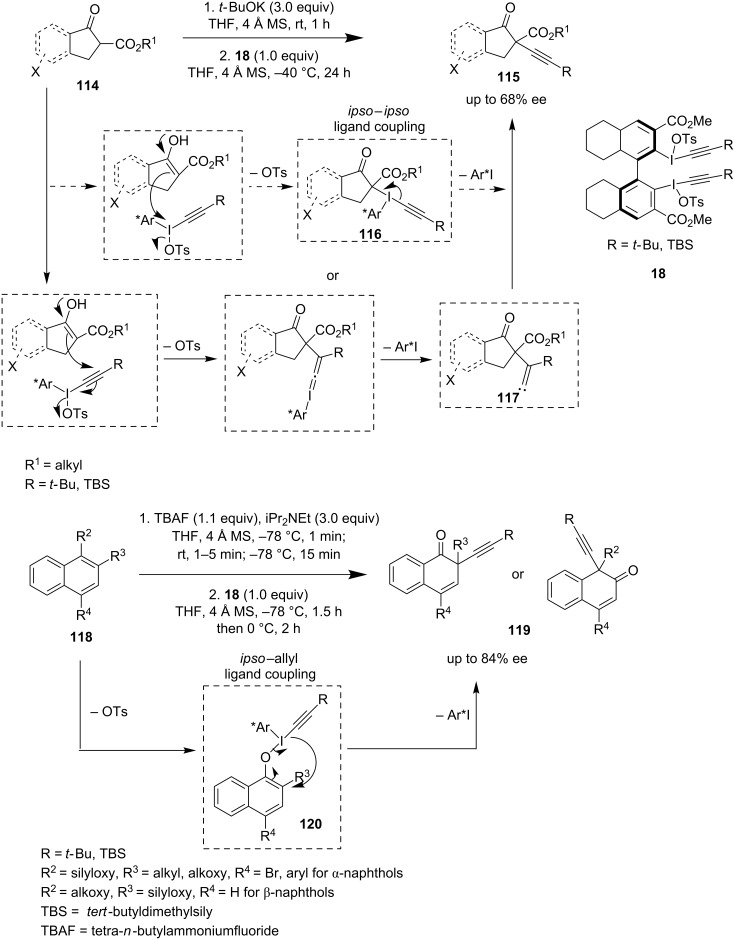
Alkynylation of β-ketoesters and dearomative alkynylation of phenols.

Pouységu and Quideau et al. prepared new axially chiral biaryl I(III) reagents **18** assembled with alkynyl ligands. They were able to achieve alkynylation of β-ketoesters **114** as well as dearomative alkynylation of phenolic derivatives **118** to obtain derivatives **115** and **119**, respectively, with decent enantioselectivity (Scheme 25) [77]. The formation of an alkylidene carbene **117** and its rapid rearrangement via 1,2-silyl shift (in case of R = silyl group) into the alkylated β-ketoesters **115** can fairly explain the reaction outcome. On the other hand, the ligand exchange/coupling sequence through the iodosyl intermediate **116** can be an alternative pathway for the formation of **115**. Likewise, a C**–**C *ipso*–allyl ligand coupling via intermediate **120** from *O*-naphtholate **118** explains the formation of product **119**.

## Conclusion

To conclude, throughout this review we have seen substantial growth in the field of chiral hypervalent iodine reagents. This review points a number of striking chiral hypervalent iodine reagents used in stoichiometric or in catalytic fashion for quite a number of useful organic transformations. Most importantly the oxidative chemistry can be done using catalytic amounts of chiral hypervalent iodine reagents in the presence of an external oxidant. For convenient reading, we have only highlighted the optimized chiral catalysts or reagents used in the mentioned transformations. We hope that these new classes of reagents can achieve synthetically more challenging and application-oriented conversions that can be applied for the total synthesis of natural products as well as in industry related to pharmaceutical and medicinal chemistry.

These environmentally friendly, cheap and readily available reagents will surely attract the attention of scientists towards a sustainable replacement of transition metals. The application of chiral hypervalent iodine reagents is expected to pave the way for new reactions and reagent design in the field of asymmetric synthesis and catalysis.

## References

[1] Willgerodt C (1886). J Prakt Chem.

[2] Zhdankin V V, Stang P J (2002). Chem Rev.

[3] Zhdankin V V, Stang P J (2008). Chem Rev.

[4] Yoshimura A, Zhdankin V V (2016). Chem Rev.

[5] Zhdankin V V (2009). ARKIVOC.

[6] Zhdankin V V (2011). J Org Chem.

[7] Yusubov M S, Zhdankin V V (2012). Curr Org Synth.

[8] Merritt E A, Olofsson B (2009). Angew Chem, Int Ed.

[9] Küpper F C, Feiters M C, Olofsson B, Kaiho T, Yanagida S, Zimmermann M B, Carpenter L J, Luther G W, Lu Z, Jonsson M (2011). Angew Chem, Int Ed.

[10] Wirth T (1999). Synthesis.

[11] Wirth T (2005). Angew Chem, Int Ed.

[12] Kitamura T, Fujiwara Y (1997). Org Prep Proced Int.

[13] Varvoglis A (1997). Tetrahedron.

[14] Moriarty R M (2005). J Org Chem.

[15] Kiprof P (2005). ARKIVOC.

[16] Ochiai M, Sueda T, Miyamoto K, Kiprof P, Zhdankin V V (2006). Angew Chem, Int Ed.

[17] Sajith P K, Suresh C H (2012). Inorg Chem.

[18] Gillespie R J, Silvi B (2002). Coord Chem Rev.

[19] Akiba K y (1999). Chemistry of Hypervalent Compounds.

[20] Zhdankin V V (2014). Hypervalent Iodine Chemistry.

[21] Parra A, Reboredo S (2013). Chem – Eur J.

[22] Berthiol F (2015). Synthesis.

[23] Pribram R (1907). Justus Liebigs Ann Chem.

[24] Imamoto T, Koto H (1986). Chem Lett.

[25] Tohma H, Takizawa S, Watanabe H, Fukuoka Y, Maegawa T, Kita Y (1999). J Org Chem.

[26] Hatzigrigoriou E, Varvoglis A, Bakola-Christianopoulou M (1990). J Org Chem.

[27] Xia M, Chen Z-C (1997). Synth Commun.

[28] Zhdankin V V, Smart J T, Zhao P, Kiprof P (2000). Tetrahedron Lett.

[29] Ladziata U, Carlson J, Zhdankin V V (2006). Tetrahedron Lett.

[30] Ray D G, Koser G F (1990). J Am Chem Soc.

[31] Altermann S M, Schäfer S, Wirth T (2010). Tetrahedron.

[32] Dohi T, Maruyama A, Takenaga N, Senami K, Minamitsuji Y, Fujioka H, Caemmerer S B, Kita Y (2008). Angew Chem, Int Ed.

[33] Dohi T, Takenaga N, Nakae T, Toyoda Y, Yamasaki M, Shiro M, Fujioka H, Maruyama A, Kita Y (2013). J Am Chem Soc.

[34] Dohi T, Sasa H, Miyazaki K, Fujitake M, Takenaga N, Kita Y (2017). J Org Chem.

[35] Boppisetti J K, Birman V B (2009). Org Lett.

[36] Fujita M, Okuno S, Lee H J, Sugimura T, Okuyama T (2007). Tetrahedron Lett.

[37] Uyanik M, Yasui T, Ishihara K (2010). Angew Chem, Int Ed.

[38] Uyanik M, Yasui T, Ishihara Y K (2010). Tetrahedron.

[39] Uyanik M, Yasui T, Ishihara K (2013). Angew Chem, Int Ed.

[40] Jain N, Xu S, Ciufolini M A (2017). Chem – Eur J.

[41] Quideau S, Lyvinec G, Marguerit M, Bathany K, Ozanne-Beaudenon A, Buffeteau T, Cavagnat D, Chénedé A (2009). Angew Chem, Int Ed.

[42] Bosset C, Coffinier R, Peixoto P A, El Assal M, Miqueu K, Sotiropoulos J-M, Pouységu L, Quideau S (2014). Angew Chem, Int Ed.

[43] Fujita M, Yoshida Y, Miyata K, Wakisaka A, Sugimura T (2010). Angew Chem, Int Ed.

[44] Wirth T (1995). Angew Chem, Int Ed Engl.

[45] Wirth T, Hirt U H (1997). Tetrahedron: Asymmetry.

[46] Hirt U H, Spingler B, Wirth T (1998). J Org Chem.

[47] Hirt U H, Schuster M F H, French A N, Wiest O G, Wirth T (2001). Eur J Org Chem.

[48] Fujita M, Wakita M, Sugimura T (2011). Chem Commun.

[49] Shimogaki M, Fujita M, Sugimura T (2016). Angew Chem, Int Ed.

[50] Röben C, Souto J A, González Y, Lischynskyi A, Muñiz K (2011). Angew Chem, Int Ed.

[51] Richardson R D, Desaize M, Wirth T (2007). Chem – Eur J.

[52] Farid U, Wirth T (2012). Angew Chem, Int Ed.

[53] Kong W, Feige P, de Haro T, Nevado C (2013). Angew Chem, Int Ed.

[54] Banik S M, Medley J W, Jacobsen E N (2016). Science.

[55] Banik S M, Medley J W, Jacobsen E N (2016). J Am Chem Soc.

[56] Woerly E M, Banik S M, Jacobsen E N (2016). J Am Chem Soc.

[57] Farid U, Malmedy F, Claveau R, Albers L, Wirth T (2013). Angew Chem, Int Ed.

[R58] 58For a racemic version of the same rearrangement: Moriarty, R. M.; Khosrowshahi, J. S.; Parakash, O. *Tetrahedron Lett.* **1985,** *26,* 2961–2964. doi:10.1016/S0040-4039(00)98592-7

[59] Brown M, Kumar R, Rehbein J, Wirth T (2016). Chem – Eur J.

[60] Qurban J, Elsherbini M, Wirth T (2017). J Org Chem.

[61] Dong D-Q, Hao S-H, Wang Z-L, Chen C (2014). Org Biomol Chem.

[62] Ochiai M, Takaoka Y, Masaki Y, Nagao Y, Shiro M (1990). J Am Chem Soc.

[63] Ochiai M, Kitagawa Y, Takayama N, Takaoka Y, Shiro M (1999). J Am Chem Soc.

[64] Jalalian N, Olofsson B (2010). Tetrahedron.

[65] Brown M, Delorme M, Malmedy F, Malmgren J, Olofsson B, Wirth T (2015). Synlett.

[66] Altermann S M, Richardson R D, Page T K, Schmidt R K, Holland E, Mohammed U, Paradine S M, French A N, Richter C, Bahar A M (2008). Eur J Org Chem.

[67] Brenet S, Berthiol F, Einhorn J (2013). Eur J Org Chem.

[68] Mizar P, Wirth T (2014). Angew Chem, Int Ed.

[69] Uyanik M, Okamoto H, Yasui T, Ishihara K (2010). Science.

[70] Wu H, He Y-P, Xu L, Zhang D-Y, Gong L-Z (2014). Angew Chem, Int Ed.

[71] Cao Y, Zhang X, Lin G, Zhang-Negrerie D, Du Y (2016). Org Lett.

[72] Levitre G, Dumoulin A, Retailleau P, Panossian A, Leroux F R, Masson G (2017). J Org Chem.

[73] Suzuki S, Kamo T, Fukushi K, Hiramatsu T, Tokunaga E, Dohi T, Kita Y, Shibata N (2014). Chem Sci.

[74] Fernández González D, Brand J P, Mondière R, Waser J (2013). Adv Synth Catal.

[75] Fernández González D, Brand J P, Waser J (2010). Chem – Eur J.

[76] Wu X, Shirakawa S, Maruoka K (2014). Org Biomol Chem.

[77] Companys S, Peixoto P A, Bosset C, Chassaing S, Miqueu K, Sotiropoulos J-M, Pouységu L, Quideau S (2017). Chem – Eur J.

